# Management and optimization of chronic renal insufficiency in the setting of kidney cancer A Systematic Review

**DOI:** 10.1590/S1677-5538.IBJU.2025.0303

**Published:** 2025-06-15

**Authors:** Jessica K Cobb, Hiroko Miyagi, Jad Chahoud, Claude Bassil, Philippe E. Spiess

**Affiliations:** 1 Thomas Jefferson University Department of Internal Medicine Philadelphia PA USA Department of Internal Medicine, Thomas Jefferson University, Philadelphia, PA, USA; 2 Department of Genitourinary Oncology and Department Onco-Nephrology H. Lee Moffitt Cancer Center Tampa FL USA Department of Genitourinary Oncology and Department Onco-Nephrology H. Lee Moffitt Cancer Center, Tampa, FL; 3 University of San Raffaele Division of Nephrology and Hypertension and Department of Medical Oncology Milan Italy Division of Nephrology and Hypertension and Department of Medical Oncology, University of San Raffaele, Milan, Italy; 4 University of South Florida Health Morsani College of Medicine Tampa FL USA University of South Florida Health Morsani College of Medicine, Tampa, FL, USA.

**Keywords:** Nephrology, Medical Oncology, Surgical Oncology

## Abstract

**Purpose:**

There is a bidirectional relationship between chronic kidney disease and the incidence of renal cell carcinoma. Despite the frequency of patients with both chronic kidney disease and renal cell carcinoma, there are limited systematic reviews detailing the nuanced treatment. This review provides comprehensive insights for clinicians for managing chronic kidney disease, and renal cell carcinoma.

**Methods and Methods:**

We reviewed published literature that examined either chronic kidney disease and renal cell carcinoma or an indirect contributor of both.

**Results:**

We compare and contrast renal cell carcinoma treatment with partial and radical nephrectomies, ablative techniques, and radiation and their impact on glomerular filtration rate, recurrence rate, and contraindications. We discuss when and how to intervene with treatment with emphasis on the delicate balance between eradicating malignancy and preserving renal function. Specifically, we detail the appropriate use of renal biopsies in incidentally discovered tumors, active surveillance, and postoperative surveillance including imaging sensitivity and specificity. We offer insight into the limitations of current systemic therapy, including renal toxicity.

**Conclusions:**

Our investigation into the intricate relationship between chronic kidney disease and renal cell carcinoma has many multifaceted challenges for both patients and healthcare providers face. This comprehensive review serves as an extensive synopsis of the current literature and offers patients the best possible long-term renal-based outcomes.

## INTRODUCTION

Chronic kidney disease (CKD) is characterized by persistent abnormalities in kidney function or structure with an estimated glomerular filtration rate (eGFR) <60 mL/min/1.73m^2^ that persist for more than 90 days. CKD has been recognized as a worldwide public health problem as its estimated global prevalence is 9.1%, impacting around 697.5 million individuals (1). CKD is a progressive condition and is typically insidious at milder stages because of the kidneys’ compensatory mechanisms; symptoms typically develop when eGFR falls below 30 mL/min/1.73m2. Reduction in eGFR correlates with increased mortality and rate of cardiovascular disease. The incidence of RCC among patients receiving dialysis is more than 3× higher than in the general population (2).

Of all cancer diagnoses, renal cell carcinoma (RCC) makes up 2.4% globally with 400,000 new cases and 180,00 deaths in 2020 (3, 4). The incidence of RCC has increased, which can partially be attributed to extensive use of abdominal imaging to assess various clinical conditions resulting in a decreased in stage of RCC at the time of diagnosis (4). The 5-year survival rates for RCC are stage-dependent, with stage 1 having a rate of 90%, stage 2 at 50%, stage 3 at 30%, and stage 4 at 5% (5). As the prevalence of RCC increases, the bidirectional relationship between CKD and RCC has become more evident (3).

**Figure 1:**
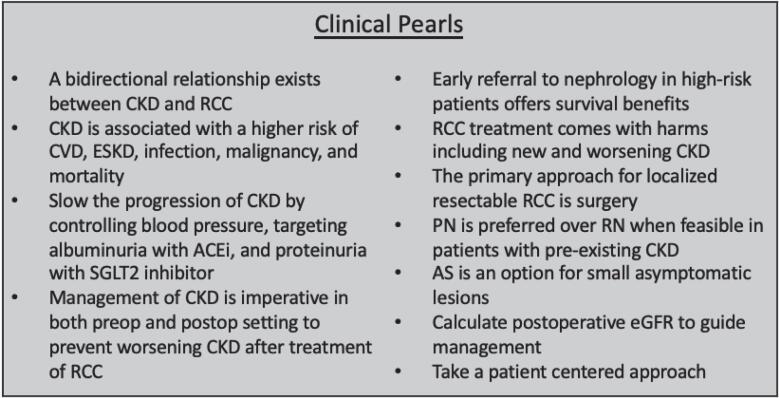
Clinical pearls in diagnosis and management.

This review explores the balance between eradicating malignancy and preserving renal function. This guide serves as a comprehensive overview of the current literature to equip providers with the necessary information to offer patients the best possible long-term outcomes (Figure-1).

Figure depicting key clinical pearls in diagnosis and management of CKD and RCC. CKD = chronic kidney disease; RCC = renal cell carcinoma; CVD = cardiovascular disease; ESKD = end stage renal disease; ACEi = angiotensin-converting enzyme inhibitors; SGLT2 = sodium-glucose co-transporter 2; preop = preoperative; postop = postoperative; PN = partial nephrectomy; RN = radical nephrectomy; AS = active surveillance; eGFR = estimated glomerular filtration ratio

## MATERIALS AND METHODS

Inclusion criteria included using keywords in PubMed from accessed from October 2023 through June 2024, considering impact factors and citation frequency. For novel ideas, the team relied on coauthors who are experts in the fields of nephrology, nephron-oncology, and urology. Exclusion Criteria excluded lower-quality publications and those that are not readily available in the English language. Studies included but were not limited to randomized control trials, observational studies, national guidelines, and systemic reviews that examined either CKD, RCC or an indirect contributor of both.

Our search strategy involved combining terms related to CKD (e.g., "postoperative eGFR") and RCC (e.g., "partial nephrectomy," "surgical CKD"). An example search string for PubMed was" ("CKD" OR "ESRD") AND ("RCC" OR "renal cancer"). The list of keywords and dates accessed is available in the supplemental data.

The electronic database used was primarily Pubmed, with supplemental Google Scholar and manual searches of the reference list. Endnote was used to facilitate correct references. No formal risk of bias assessment was completed; however, overt biases and heterogeneity were noted in text when appropriate.

Titles and abstracts identified through the search were screened independently by two reviewers and then further examined by five additional reviewers. Full-text articles of potentially relevant studies were assessed for eligibility based on the inclusion criteria. No disagreements arose during the selection process.

## RESULTS

### Preoperative counseling for RCC

#### Management options for RCC

RCC treatment is stage-dependent and may include partial nephrectomy (PN) or radical nephrectomy (RN), ablative techniques, stereotactic body radiation therapy (SBRT), and systemic therapy. Nephrectomy, either partial or radical, is the definitive treatment for RCC. A risk benefit analysis between RN and PN needs to be considered as RN offers improved 5-year cancer-specific survival rates, but PN preserves more renal function; therefore, PNs are preferred over RNs for patients with preexisting CKD and proteinuria, while RN is preferential for patients with concerning oncologic potential (6).

Ablative techniques, such as cryoablation, radiofrequency ablation, and microwave ablation, offer alternative approaches available for small renal masses that are often less invasive and offer greater nephron sparing than conservative treatment (6). While cryoablation's impact on eGFR is comparable to PN (6% decline at 2 years vs 5-8.4% at 1-3 years), the recurrence rate for PN is much preferential (3.2% global recurrence with mean time to recurrence at 47 months) to cryoablation (5% local recurrence at 6-18 months) (Table-1) (6–13). However at this time ablative procedures should only be utilized for patients with stage T1 RCC due to greater local recurrence rate (14).

**Table 1:** Table demonstrating impact on eGFR and recurrence rate by treatment. (7–13, 49)

Treatment Type	Impact on eGFR	Recurrence Rate	Mean time to recurrence (months)
RN	32%	11%	100.8
PN	5-8.4%	3.2%	47
Cryoablation	6%	5% *	6-18 *
RFA	3.7%	9.7%	15.6

* Only local recurrence reported.

eGFR = estimated glomerular filtration rate; RN = radical nephrectomy; PN = partial nephrectomy; RFA = radiofrequency ablation.

Although RCC has historically been considered resistant to radiation, technological advances in radiation therapy have allowed SBRT to be efficacious for local tumors or metastatic sites, offering a noninvasive approach without significant treatment-related toxicity. Recently a multicenter phase II trial with SBRT demonstrated with a mean follow-up time of 42 months with 100% cancer-specific survival with a mean decrease eGFR of 14.6 mL/min/1.73^2^ (n=70) (15). The 2025 NCCN guidelines now list SBRT as an option for non-optimal surgical candidates (14).

#### Risk of post-treatment progressive CKD

Surgical removal of RCC has excellent 5-year cancer-specific survival rates (87% to 90% after PN and 96.7% after RN) (16). However, PN and RN independently contribute to the post-surgical increased risk for the development and progression of CKD.

A randomized phase III trial demonstrated patients with T1 RCC and a normal contralateral kidney did not have overall survival (OS) advantages or improvements in rates of kidney failure (eGFR <15mL/min1.73m2) with PN compared to RN (17). However, study limitations include small sample size, poor accrual, and substantial loss to follow-up. Data from a systematic review and meta-analysis for T2 or higher masses have shown improved preservation of kidney function and lower decline in renal function after PN than RN (16). Patients who received PN experienced improved OS (n=5,056; HR: 0.77; 95% CI: 0.65-0.90; p = 0.002; I2=0%). Based on the evidence that renal function is better preserved following PN than RN, there has been growing interest in the use of PN to treat larger masses (Table-1).

In patients undergoing PN for a renal mass in a solitary kidney, the main factor determining postoperative renal function is the parenchymal volume preservation (18). Other factors have been demonstrated to correlate with postoperative renal function, including AKI, type/duration of ischemia, complexity of tumor, and comorbidities; however, their influence was less than that of parenchymal volume preservation (n=841, r = 0.84, p < 0.001).

#### Medical vs surgical CKD

Though CKD secondary to medical causes (CKD-m) is associated with an annual reduction in renal function of 2% to 5%, surgically-induced CKD (CKD-s) related to the removal of functioning nephrons does not have the same decline (0.7%/ year decline)(n=44,808, p <0.001) (19). The distinct absence of ongoing decline has been attributed to a lack of so-called "drivers of CKD," most notably diabetes and hypertension (Table-2) (20). Postoperative data has shown that patients with both CKD-m and CKD-s experienced the highest overall mortality (19). Furthermore, the risk of death after surgery was significantly higher for patients with preoperative CKD-m than patients with normal preoperative renal function (2.7×, 3.5×, and 4.4× higher for stage 3, 4 and 5 CKD respectively)(n=4,180, CI 1.8-5.0; CI 2.4-5.9; CI 2.8-7.0 respectively) (20). Among patients without CKD-m, preoperative eGFR was not a predictor of OS. The survival curve for patients who developed CKD-s was similar to those with normal postoperative eGFR levels, as long as new baseline eGFR is > 45 mL/min/1.73^2^. If the GFR fell below this threshold the mortality and risk of functional decline increased significantly. This suggests that patients with CKD-s experience much better outcomes than those with CKD-m, and the two disease subtypes should be treated as separate entities.

**Table 2:** Relative risk of CKD by risk factor. (47, 48)

Risk Factors	Relative Risk
HTN only^1^	2.0 (95% CI 1.8-2.2)
HTN, HLD, and high BMI	2.6 (95% CI 2.2-2.9)
HTN and DM	3.3 (95% CI 2.9-3.8)
HTN, HLD, high BMI, and DM	5.5 (95% CI 4.9-6.2)
Obesity (BMI >30 kg/m^2^)	1.77 (95% CI 1.47-2.14)
Smoking^2^	1.52 (95% CI 1.13-2.06)
Physical inactivity^3^	2.14 (95% CI 1.39-3.30)
Obesity, smoking^2^, and physical inactivity^3^	5.10 (95% CI 2.36-11.01)

CKD = chronic kidney disease, HTN = hypertension; CI = confidence interval; HLD = hyperlipidemia; BMI = body mass index; DM = diabetes mellitus.

1 = without other risk factors.

2 = >25 pack-years.

3 = no or some physical activity in leisure time.

#### Predicting postoperative renal function

Given the increased risk of CKD following RN compared to PN, careful preoperative renal function and contralateral renal status is imperative. A cutoff line exists for estimated postsurgical baseline GFR above 45 mL/min/1.732; if estimated below the cutoff, even if RN is preferred, then PN is recommended. This cutoff is strongly associated with improved survival outcomes (n=1,479; HR: 2.8; p<0.001) (6, 19, 21). Additionally, actual postoperative eGFR <65mL/min/1.73m^2^ were associated with increased cancer-specific mortality for PN or RN with a significant increase in the subdistribution hazard ratio for every 10mL/min reduction in eGFR (n=3,457; HR: 1.25; 95% CI:1.07–1.44, p = 0.003) (22). Therefore, there has been increased research in developing tools to predict postoperative eGFR, called new baseline GFR (NBGFR).

Currently, the most reliable way to predict NBGFR after RN involves a model to determine parenchymal volume analysis (PVA) based on preoperative GFR, split renal function (SRF), and renal function compensation (RFC). Historically, SRF has been determined by nuclear renal scans; however, PVA via software analysis has been shown to provide more accurate and precise SRF and preclude the need for renal scans (n=187; r=0.85; p<0.05) (21, 23).

### Competing risks

#### Life-preserving and life-limiting dialysis

The incidence of RCC among patients receiving dialysis is 3× higher than in the general population (n=831,804; SIR 3.6; CI 3.5 to 3.8; P < 0.0001) (2). This risk, along with carcinoma aggressiveness, further increases after a decade of dialysis use; therefore, periodical screening for RCC should be considered. There are not current guidelines for this population, as cancer screening guidelines are created to improve health outcomes via early detection; however, the 5-year survival rate for patients after initiation of maintenance dialysis is approximately 40%. Given the limited life expectancy of many of these patients, screening needs to be individually tailored to those who are most likely to benefit from early detection, such as in younger patients with a longer anticipated life span (24).

### Systemic treatment of RCC

Systemic treatments, utilized in the context of metastatic RCC (mRCC), unresectable RCC, or adjuvant therapy, include immune checkpoint inhibitors (ICIs) and targeted molecular therapy (e.g., VEG-F-TKIs and mTOR inhibitors). Though these systemic treatments have offered improvements in OS, they may be associated with nephrotoxicity and worsening renal function. All patients should be closely followed for nephrotoxicity after treatment with systemic therapy.

ICI associated AKI (ICI-AKI) is a rare but potentially serious complication, with a meta-analysis demonstrating an incidence of 2.2% (n=11,482; 95% CI 1.5-3.0%; I^2^=68%); a multicenter study indicated that up to 15% of patients who develop AKI will not experience renal recovery (n=138; HR 3.91; 95% CI 1.22-12.59) (25, 26). For patients that experience ICI-AKI, clinicians should hold ICIs and initiate treatment with glucocorticoids. Patients may be rechallenged with ICI after kidney function improves. An observational study noted 84% of patients with ICI-AKI, rechallenged did not redevelop ICI-AKI (n=429) (27).

Anti-VEGF agents and TKIs are associated with proteinuria and rarely associated with nephrotic syndrome. A meta-analysis demonstrated proteinuria with VEGF-TKIs as 18.7% and 2.4% for all-grade and high-grade proteinuria respectively (n=6,682; all grade: 95% CI, 13.3%-25.6% Q = 400.96; *P<0.001; I*^2^ = 94%; high grade Q = 72.46; *P<0.001; I*^2^ = 64%; 95% CI, 1.6%-3.7% respectively); the severity of proteinuria is increased in patients with preexisting renal disease (28–31). Stopping the offending agent often results in significant reduction in proteinuria, although persistence is common which may be treated with angiotensin-converting enzyme inhibitors (ACEi) and angiotensin-receptor blockers (ARB) (32).

Of note, the risk of CKD in the setting of adjuvant setting is likely higher than reported as many trials exclude patients with low eGFR (e.g. KEYNOTE 564 excluded patients with eGFR < 40 mL/min/1.73m^2^), while this is not a guideline recommended cutoff for pembrolizumab (32). Additionally, patients in this trial had labs every 3 weeks monitoring for urinary protein/creatinine ratio with clear cut off guidelines, while urinalysis is more commonly ordered every 6-8 weeks with termination of therapy at physician's discretion (33).

Risk stratification should be completed prior to starting chemotherapy. International Metastatic Renal Cell Carcinoma Database Consortium (IMDC) is a risk model for mRCC which uses clinical and laboratory parameters to risk stratify the patient (intermediate/poor versus favorable) (34, 35). The NCCN guidelines recommend preferred regimen based on the favorability determined by the IMDC (14).

### Postoperative treatment surveillance

#### Surveillance guidelines & impact on renal function

Posttreatment surveillance imaging allows for early local recurrence detection and metastases identification, improving survival rates with timely re-intervention (Table-3). Long-term follow up is imperative as 30% of recurrences are discovered over 5 years after treatment (36). However, contrast enhanced imaging is not without possible renal implications.

**Table 3:** Diagnostic imaging modalities for patients with respective sensitivity and specificity (49).

Imaging Type	Sensitivity	Specificity
Contrast enhanced CT	88%	75%
Unenhanced US	56%	71%
Contrast enhanced US	93%	72.5%
Contrast enhanced MRI	87.5%	89%
FDG/PET	88%	87.5%

CT = computed tomography; US = ultrasound; MRI = magnetic resonance imaging; FDG = fluorodeoxyglucose; PET = positron emission tomography.

Per the American College of Radiology (ACR) Committee on Drugs and Contrast Media, IV iodinated contrast media is not an independent nephrotoxic risk factor in those with a stable baseline eGFR ≥45 mL/min/1.73m^2^ (37). In patients with eGFR 30-44 mL/min/1.73m^2^ it is either rarely or not nephrotoxic. However research on those with eGFR <30 mL/min/1.73m^2^ have conflicting results. Two studies which were propensity-score matched showed IV iodinated contrast material as an independent nephrotoxic risk factor while two others found no such evidence. Studies in support demonstrated that these patients have a 3x increased risk of iodinated contrast induced AKI (CI-AKI) (38–42). Persistent renal damage from CI-AKI is proposed to occur among 18.6% of patients with moderate to severe baseline renal impairment (n=3,986) but only 1.2% of the general population (n=4,418) (43, 44).

Anuric patients with ESRD may receive IV iodinated contrast; however, oliguric patients on dialysis should be treated as similar to patients with eGFR <30 mL/min/1.73m2, and a contrast risk-benefit analysis should be considered (45). While there is data demonstrating a dose-toxicity relationship, if the risk-benefit ratio favors contrast-enhanced imaging, it is not recommended to reduce contrast doses in attempts to mitigate risk of CI-AKI as this may result in suboptimal imaging (45).

### MRI Contrast

MRI imaging with gadolinium contrast is preferential for patient who cannot tolerate any conventional contrast (36). For patients with ESRD on chronic dialysis, it is recommended to undergo GBCA-enhanced MRI before regularly scheduled dialysis although the evidence is lacking proving improved safety as dialysis does not improve GBCA clearance (45). Patients’ ineligible for CT contrast and MRI should be considered for contrast enhanced ultrasound.

## DISCUSSION

The bidirectional relationship between CKD and RCC is established, and they are both relatively frequent diagnoses; however, systematic reviews detailing the nuanced treatment with respect to both are lacking.

We discussed the pros and cons for PN, RN, ablative techniques, SBRT, and systemic therapy. Each has strengths and weaknesses, and the risk of further renal damage must be part of the patient/provider discussion. RN offers improved 5-year cancer-specific survival rates, while PN preserves more renal function. Ablative techniques offer greater nephron sparing than conservative treatments at the cost of increased recurrence rates, and therefore, they are only recommended for low-stage RCC.

A cutoff line exists for estimated postsurgical baseline GFR above 45 mL/min/1.732, which is strongly associated with improved survival outcomes. Currently, providers should predict NBGFR with PVA, and RNs are not advised if NBGFR is less than 45 mL/min/1.732.

Providers should monitor patients on systemic therapy for renal toxicity, which may necessitate stopping the offending agent. Providers should be wary that the risk of CKD in the adjuvant setting is likely underreported, as many trials’ requirements do not reflect real-world conditions (excluding patients with low eGFR and increased post-treatment lab frequency). To guide the selection of systemic therapy and estimate the median survival of patients with mRCC, providers should use risk stratification with IMDC.

Limitations of our systematic review may include publication bias, heterogeneity, possibly poorer quality of studies than initially anticipated, and time lag bias.

## CONCLUSIONS

In conclusion, our investigation into the intricate relationship between CKD and RCC has many multifaceted challenges for both patients and healthcare providers face. When considering treatment modalities for RCC providers must consider the delicate balance between eradicating malignancy and preserving renal function. An individualized approach, coupled with ongoing research to refine guidelines and strategies, is crucial for optimizing patient outcomes.
